# Effect of Psyllium Husk Addition on the Structural and Physical Properties of Biodegradable Thermoplastic Starch Film

**DOI:** 10.3390/ma15134459

**Published:** 2022-06-24

**Authors:** Karolina Beer-Lech, Anna Skic, Kamil Skic, Zbigniew Stropek, Marta Arczewska

**Affiliations:** 1 Department of Mechanical Engineering and Automatic Control, University of Life Sciences in Lublin, 28 Głęboka St., 20-612 Lublin, Poland; anna.skic@up.lublin.pl (A.S.); zbigniew.stropek@up.lublin.pl (Z.S.); 2 Institute of Agrophysics, Polish Academy of Sciences, 4 Doświadczalna St., 20-290 Lublin, Poland; k.skic@ipan.lublin.pl; 3 Department of Biophysics, Faculty of Environmental Biology, University of Life Sciences in Lublin, 13 Akademicka St., 20-950 Lublin, Poland; marta.arczewska@up.lublin.pl

**Keywords:** biocomposites, thermoplastic starch film, psyllium husk, microstructure, mechanical properties, barrier properties

## Abstract

The research subject was the analysis of the microstructure, barrier properties, and mechanical resistance of the psyllium husk (PH)-modified thermoplastic starch films. The tensile tests under various static loading conditions were not performed by researchers for this type of material before and are essential for a more precise assessment of the material’s behavior under the conditions of its subsequent use. The film samples were manufactured by the casting method. PH addition improved starch gelatinization and caused a decrease in failure strain by 86% and an increase in failure stress by 48% compared to pure films. Fourier transform infrared spectroscopy results showed the formation of additional hydrogen bonds between polysaccharides in starch and PH. An increase in the number of hydrophilic groups in the modified films resulted in a faster contact angle decrease (27.4% compared to 12.8% for pure ones within the first 5 s); however, it increased the energy of water binding and surface complexity. The modified films showed the opacity at 600 nm, 43% higher than in the pure starch film, and lower transmittance, suggesting effectively improving barrier properties to UV light, a potent lipid-oxidizing agent in food systems.

## 1. Introduction

Polymer-based materials made of biodegradable raw materials are gaining more and more advantage over traditional synthetic materials due to their environmental friendliness and easy availability [1]. The increase in global demand for these types of materials has been caused by the introduction of a legislative requirement to withdraw disposable food packaging made of synthetic materials [2]. Polymer plastics are so problematic that even their selective collection does not help to reduce their quantity. Many of them are never deposited in landfills, pollute the ecosystem, and are a true killer of the ocean fauna. According to the European Commission, more than 80% of marine litter is plastic. Products covered by the new rules account for 70% of all marine litter items (details are available in [3]). Using edible films and coatings in food packaging is a beneficial way to reduce the amount of synthetic polymeric materials and to extend the shelf life of products.

Starch is recognized as an important polysaccharide polymer due to its ease of forming a continuous matrix, low manufacturing cost, easy renewability, and abundance [4,5,6]. Native starch is a semi-crystalline material composed mostly of amylose and amylopectin polymers, whose structures include glucose monomer units [6,7]. Strong intra- and inter-molecular hydrogen bonds between hydroxyl groups of starch polymers make them decompose instead of melting upon heating [8]. The thermoplastic properties of starch are revealed in the presence of an appropriate plasticizer and water, which trigger the process of transformation of semi-crystalline starch granules into a homogeneous thermoplastic starch structure (TPS). Starch-based edible films can be synthesized by two methods: the casting and extrusion techniques, also known as wet and dry methods [9]. Previous studies have documented that the type of plasticizer has a large impact on the mechanical, thermal, and barrier properties of starch-based films [10]. Plasticized starch films showed more flexibility and workability due to weakening the intermolecular forces between the polymer chains, thereby increasing the molecular volume [11,12]. Among the plasticizing agents, glycerol is characterized by high compatibility with amylose that can strongly affect the functional properties of starch-based coatings and films by interfering with amylose chain packing [13].

However, despite many studies on TPS-based films, there are still technological application problems related to their low mechanical strength [14] and high water vapor permeability (WVP). Pure native starch films are brittle and tend to absorb large quantities of water at elevated relative humidity (RH) conditions (because of their inherent hydrophilic nature) compared with synthetic polymers, such as polyethylene. To increase the functional properties of thermoplastic starch films, various functional additives in the form of fibers, seeds, protein additives, hydrophobic biodegradable polymers, ceramic additives, or waxes are used [15,16,17,18].

One of the additives that have recently become of special interest to scientists is psyllium plantain and its various physical forms, such as seeds, husks, flour, or psyllium husk gum [19,20]. Psyllium is obtained from the seeds of the plant genus *Plantago* and *Plantago ovata*, which has more than 200 species. Among its many physical forms, psyllium husk has a wide nutritional value and is used for manufacturing psyllium products because of its low cost, non-toxicity, biodegradability, and biocompatibility [21,22]. The bioactive component of psyllium is composed of a highly branched polysaccharide-arabinoxylan, which contains both (1–4) and β-(1–3) glycosidic linkages in the xylan backbone [23]. Biodegradable films with improved physical, mechanical, and barrier properties have already been investigated using composites containing native starch, whey protein concentrate, and psyllium husk in the context of designing films with specific spectral characteristics [24,25]. Some authors focus on films manufactured by extraction of psyllium hydrocolloid and preparation of an edible film directly from PH [19], while others use PH as a small additive to starch-based films [24]. It has been reported that the application of psyllium husks as a filler in the polymer matrix resulted in reduced free volume and moisture diffusion, which improved the water vapor barrier properties of the modified starch films [26,27]. Most of the authors mainly present the results focusing on water vapor permeability studies combined with FTIR analysis and very little microstructure analysis. Nevertheless, psyllium seeds seem to be a promising raw material for producing edible films or coatings [1]. However, the extended investigation of mechanical properties is still rarely reported for these films.

To the best of our knowledge, the analysis of the mechanical properties of thermoplastic starch films with psyllium husk addition, in terms of tensile strength at different speeds, has not been reported in the literature. This limits the considerations to only a small range of static loads. Extending the analysis of mechanical resistance of the PH-based films under various static loading conditions combined with a detailed analysis of the structure will allow for a more precise assessment of the material’s behavior under the conditions of its subsequent use. In the available works, little attention is also paid to the analysis of sorption isotherms. These tests are aimed at presenting the relationship between water content and water activity of any material at a given temperature. Water activity is then defined as the ratio of water vapor pressure above the material to the vapor pressure of pure water at the same temperature. Sorption isotherms provide important information on the product stability in relation to microbial and enzyme activity [28], and for that reason sorption isotherms of foods and agricultural products have been extensively studied.

The objective of the present work is to evaluate the physical, mechanical, and barrier properties, together with a detailed analysis of the microstructure of films based on thermoplastic starch with psyllium husk addition. The surface parameters morphology and wettability were analyzed by atomic force microscopy (AFM) and contact angle measurements. The barrier properties were studied by determining water vapor permeability (WVP) and the moisture sorption isotherms. Tensile tests analyzed the mechanical properties under various static loading conditions. Fourier transform infrared spectroscopy (FTIR) and scanning electron microscopy (SEM) have been effectively used to gain insight into physical properties that can enrich the knowledge of interactions between matrix components and facilitate the future design of films with specific structural and barrier properties. The obtained results are promising for improving the mechanical and UV-barrier properties of starch films with PH, which is of particular interest for their application in the food packaging and coatings industry.

## 2. Materials and Methods

### 2.1. Materials

Potato starch needed to prepare the film was purchased from PPZ Trzemeszno (Trzemeszno, Poland) and psyllium husk from Witpak (Kielce, Poland). Glycerol 99.5% (Stanlab, Lublin, Poland) was used as the plasticizer.

### 2.2. Preparation of the Films

**Figure 1:**
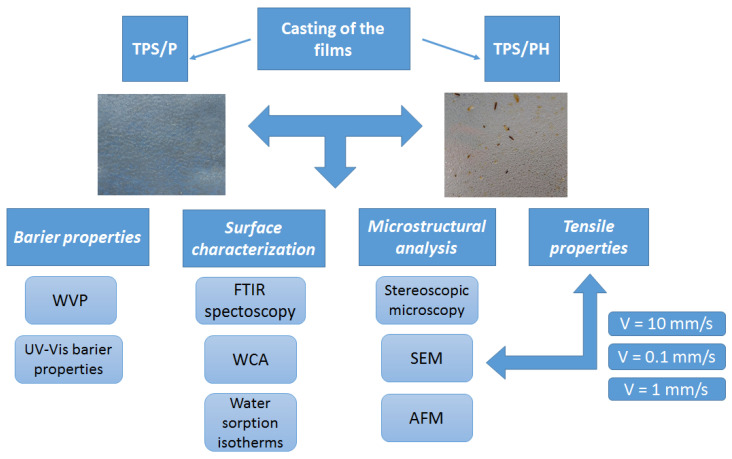
The scheme of the experimental design.

The procedure of casting thermoplastic starch films without psyllium husk (pure TPS/P) and with the addition of psyllium husk (TPS/PH) was the same and based on [29], with some modifications. Distilled water was used to prepare film-forming solutions. Potato starch and psyllium husk were added to distilled water at a concentration of 4.3% (*w*/*w*) for potato starch and 1% (*w*/*w*) for psyllium husk, respectively. The solution was heated to 80 °C under constant stirring (300 rpm) using a magnetic stirrer (Steinberg SBS-MR-1600/1T (Steinberg Systems, Zielona Góra, Poland) for 30 min. Subsequently, the solution was cooled to 40 °C and glycerol was added in the amount of 1.3% (*w*/*w*). The entire solution was mixed using an ultrasonic homogenizer TF-650N (Tefic Biotech CO., Beijing, China) for 50 min. The mixtures were poured into 200 mm × 200 mm acrylic glass molds and dried in a KBC-65 thermal research chamber (WAMED, Warszawa, Poland) at 35 °C for 20 h. After removal from the molds, the films were conditioned at the temperature of 22 °C and 40% relative humidity (RH) for 24 h. A detailed description of the experimental procedures can be tracked in Figure 1. The thickness of each film was measured with a digital micrometer with an accuracy of ±1 µm just after it was peeled from the acrylic glass mold. Thickness measurements of five locations were taken for each film (one in the center of the film and four around the perimeter) in at least ten replications. For calculations, the mean value was used (Table 1).

### 2.3. Microstructural Analysis

#### 2.3.1. Stereoscopic Microscopy

The microstructure of the samples was observed using a Nikon SMZ18 stereoscopic microscope (Nikon, Tokyo, Japan) equipped with a DS-Fi3 digital camera (Nikon, Tokyo, Japan) and NIS-Elements BR image analysis software.

#### 2.3.2. Scanning Electron Microscopy (SEM)

The microstructure of the surface and fractures of the samples were observed with an electron microscope, Phenom ProX (Thermo Fisher Scientific, Waltham, MA, USA). Before SEM imaging, they were mounted without pre-treatment onto aluminum specimen stubs, using high-purity conductive double-sided adhesive carbon tabs. SEM images were collected in SE mode with accelerating voltages of 10 kV.

#### 2.3.3. Atomic Force Microscopy (AFM)

Surface topography of the films was investigated with atomic force microscopy with the use of an AFM NTEGRA Prima (NT-MDT BV, Apeldoorn, The Netherlands) in semi-contact mode, using silicon cantilever NSG30 (NT-MDT BV, Apeldoorn, The Netherlands) with an average resonant frequency of 300 kHz. For each sample, 5 different regions, 10 × 10 μm^2^ in size, were investigated with 1 Hz scanning frequency and a resolution of 512 × 512 pixels per image. Measurements were carried out at room temperature, under atmospheric condition, and at the relative humidity of 30%. AFM height and deflection images were recorded. Images were initially processed using plain transformation, and subsequently, surface roughness parameters were determined using NT-MDT software application (NT-MDT BV, Apeldoorn, The Netherlands). The arithmetical mean deviation of the roughness profile (R_a_), root-mean-square deviation of the roughness profile (R_q_), the maximum peak height of the roughness profile (R_p_), and the maximum valley depth of the roughness profile (R_v_) were subjected to analysis.

### 2.4. Surface Characterization

#### 2.4.1. Fourier Transform Infrared Spectroscopy (FTIR)

Mid-infrared absorption spectra were obtained with attenuated total reflectance-Fourier transform infrared spectroscopy (ATR-FTIR) using IRSpirit (Shimadzu, Kyoto, Japan) equipped with a DLATGS detector. The measurements were performed in ATR mode using the QATR™-S Single-Reflection Accessory with a Diamond Crystal (Shimadzu, Kyoto, Japan). Film samples were placed directly onto the crystal with a contact area diameter of 1.8 mm and pressed against its surface with a clamp mechanism. Spectra were recorded in the range of 4000–500 cm^−1^ with 36 scans at a resolution of 4 cm^−1^ from 5 locations for each film (1 in the center of the film and 4 around the perimeter). The spectra were baseline-corrected and vector-normalized; then, for each spectrum, ten individual samples were averaged. Spectral and data analysis were performed using the Grams/AI 8.0 software (Thermo Scientific, Waltham, MA, USA).

#### 2.4.2. Water Contact Angle Measurements (WCA)

Water contact angle (wettability) measurements were carried out using a goniometer (Drop Shape Analysis System DSA100, Krüss, Germany). The measurement device included a camera for recording and image acquisition. Determination of contact angles was based on the shape of a drop settled on the smooth sample surface. Dynamic drop shape changes were recorded with a rate of 25 frames per second. Distilled water in a volume of 2 µL was used as the standard liquid. Due to the initial oscillation of deposited drops, a stable measurement of the contact angle was possible only after the kinetic energy dissipated. Water drops were stable after 1–2 ms. Contact angle measurements were continued until the maximum time of 20 s was reached. Additionally, for selected time intervals, changes in parameters such as drop volume (V) and the surface of drop contact with starch films (S) were determined. Each measurement was carried out in three replications under steady temperature (20 °C) and relative humidity conditions (RH = 45%).

#### 2.4.3. Water Sorption Isotherms

The study of the surface properties of samples was based on water vapor isotherm measurements. Isotherms were measured by the gravimetric method under constant temperature conditions of 20 °C. Particular values of water activity were obtained by using sulfuric acid with gradually decreasing (adsorption isotherm) and increasing (desorption isotherm) concentrations. The samples were equilibrated at each point for two days. The amount of adsorbed water vapor at a given water activity was calculated from the difference in weight between the sample with adsorbed water and the dried sample. From the experimental data, specific surface area (SSA), average adsorption energy (E_av_), and surface fractal dimension (D) were calculated. The SSA was calculated from the linear form of the standard BET equation [30]. Calculations of E_av_ were based on the theory of adsorption on heterogeneous surfaces presented in the work by Józefaciuk et al. [31]. Average adsorption energies were expressed as dimensionless energies, showing an excess of adsorption energy over condensation energy of adsorbate in the units of RT. The estimation of D and adsorption regime type was based on the Frenkel–Hill–Halsey (FHH) scaling law described in detail by Sokołowska et al. [32].

### 2.5. Barrier Properties

#### 2.5.1. Water Vapor Permeability (WVP)

Water vapor permeability (WVP) is the rate of water vapor transmission (WVTR) through a unit area of flat material of unit thickness induced by the unit vapor pressure difference between two surfaces, under specified humidity conditions of 75% [33]. WVP of biodegradable films was determined using the gravimetric modified cup method according to [34], with some modifications [22,23]. Round film samples were sealed to the top of glass weighing vessels (φ 30 mm) containing anhydrous calcium chloride (CaCl_2_ 0% RH as a desiccant). Parafilm was used to tightly secure the edges of the vessel. The initial weight of the cups was recorded, and the cups were placed inside an environmental chamber with air temperature of 21 °C. The chamber was maintained at 75% RH using saturated sodium chloride solution to maintain the same RH gradient across the film. The test lasted for 24 h. The weight change of the vessels with films was checked at regular time intervals (of about 1 h) and the weight change was plotted as a function of time. The slope was calculated by the linear regression method and the WVTR was calculated from the slope of the straight line divided by the transfer area using Equation (1) [27]:

(1) $$\mathrm{WVTR}=\frac{1}{A}\left(\frac{\Delta m}{t}\right)$$

The water vapor permeability (WVP) (g m^−1^ s^−1^ Pa^−1^) of the films was calculated using Equation (2) [27]:

(2) $$\mathrm{WVP}=\frac{\mathrm{WVTR}\cdot e}{S\cdot \left(R_{1}-R_{2}\right)}$$

In the above equations, ∆m is the weight change of the permeation vessel (g), t is the test time (s), A is the effective area of films (m^2^), S is the saturation vapor pressure of water (Pa) at the test temperature, R_1_ and R_2_ are the relative humidity values of the external and internal environment of the permeation vessel, and e is the thickness of the film sample (m).

#### 2.5.2. UV-Vis Barrier Properties (Opacity and Light Transmittance)

The light transmission and the transparency of the film were evaluated in film specimens of 10 × 40 mm^2^ in dimension. The specimens were placed in the aperture of a double-beam UV-Vis spectrophotometer (Cary 300 Bio by Varian) and transmittance was measured in the range of 200–800 nm.

The transparency of the O_600_ film was calculated from Equation (3) [35]:

(3) $$O_{600}=\frac{\log T_{600}}{e}$$

where O_600_ is transparency (AU/mm), T_600_ is transmittance at a wavelength of 600 nm (%), and e is the thickness of the sample (mm).

### 2.6. Tensile Properties

Tensile tests of the films, under quasi-static load conditions, were carried out using a TA.HD plus texture analyzer (Stable Micro System, Godalming, UK), equipped with Exponent 6.1.16.0 software to analyze the results, at three measuring head speeds of 0.1, 1, and 10 mm/s. Load cell capacity was 30 kg. The samples were mounted on the testing machine using A/HDT jaws (Stable Micro System, Godalming, UK) and had the shape of cuboids of constant length and width, 200 and 40 mm, respectively. The measuring length of the samples was 130 mm. The tensile test was carried out until the sample was destroyed, which resulted in a zero value of the force response and the return of the measuring head to its original position. The tests were performed at a sampling frequency of 100 Hz. For each deformation velocity, ten repetitions were performed. Tests were conducted at 24 °C and 45% RH.

During the tensile test, the force response and elongation of the sample were recorded. The following mechanical properties of the films were determined: maximum force (F_max_), failure stress (σ_f_), and failure strain (ε_f_). The elasticity modulus was determined from the slope of the linear part of the stress–strain curve.

### 2.7. Statistical Analysis

Statistical analysis was performed with Statistica, version 13.1 (TIBCO Software Inc., Palo Alto, CA, USA). A one-way analysis of variance (ANOVA) followed by Tukey’s multiple comparison tests were carried out to detect significant differences in the properties of the films. The significance level used was 0.05.

## 3. Results and Discussion

### 3.1. Microstructural Analysis

**Figure 2:**
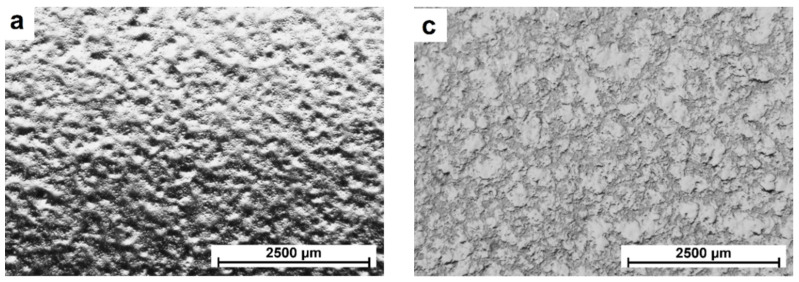

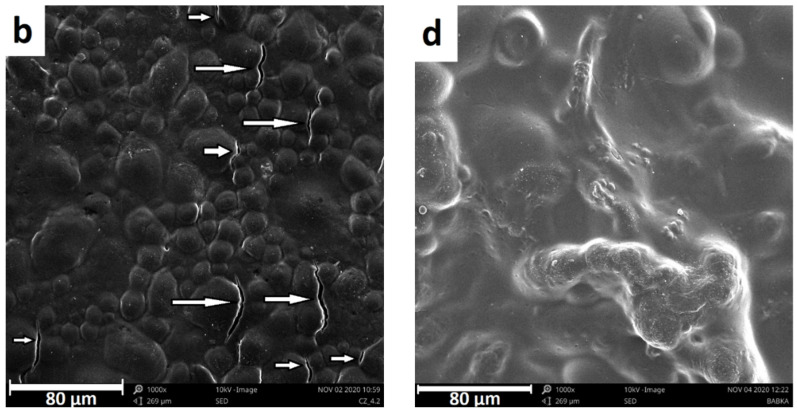
SEM images of the surfaces of TPS/P (**a**,**b**) and TPS/PH (**c**,**d**) films.

Figure 2 shows representative images of the microstructure of the tested films (TPS/P, Figure 2a,b; TPS/PH, Figure 2c,d) using a stereoscopic microscope as well as a scanning electron microscope (SEM). The films made of thermoplastic starch (TPS) presented a more compact structure compared to films prepared with the addition of psyllium husk (TPS/PH). Stereoscopic microscope observations revealed that structures of both kinds of films were free of visible pores and cracks. On the other hand, SEM observations (Figure 2b) show that the surface of TPS/P films had heterogeneous morphologies with many “small round granules” (partly marked by arrows), which can be attributed to incompletely gelatinized starch. Similar results were also obtained by Cheng et al. for films made using an extrusion process [36,37]. The authors put this phenomenon down to the low temperature during extrusion. However, there were also studies by other researchers in which starches were fully gelatinized in the blown process with higher temperature (the highest processing temperature reaches 170 and 155 °C, respectively) [38,39]. Additionally, several obvious microcracks can be observed on the surface of TPS/P films, which had been previously reported and explained as probably caused by the electron beam during SEM observation [40]. The addition of psyllium husk to TPS film (TPS/PH film) resulted in a more uniform structure with fewer “round granules” and without microcracks. This implies that psyllium husk promoted starch gelatinization. A similar effect was achieved in [36] for TPS films with ε-PL addition. The authors indicated that it is caused by hydrogen bonds’ interaction with starch granules under high shear and temperature conditions, which leads to a reduction in the intermolecular forces along starch chains and the disruption of starch granules. The formation of additional intermolecular hydrogen bonds in the TPS/PH samples was confirmed by FTIR analysis, as described in Section 3.2.1.

**Figure 3:**
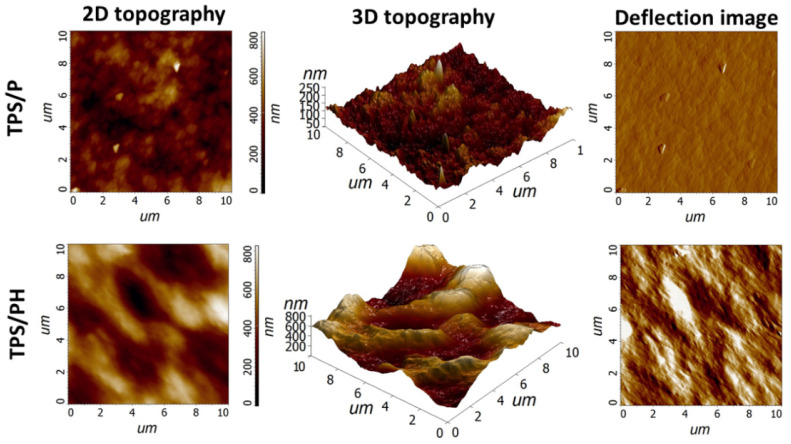
Atomic force micrographs of surface topography and deflection images of TPS/P and TPS/PH films.

Figure 3 shows the 2D and 3D topography of the samples and the corresponding deflection images. TPS/P films were characterized by a flat surface with an average roughness (R_a_) of 22.15 nm and with an average maximum peak height (R_p_) of 82.91 nm (Table 1). A similar value of R_a_ was obtained in [41] for films made from corn starch. The addition of PH caused an increase in calculated roughness parameters. The TPS/PH film surface was more irregular, with high peaks and deep valleys. R_a_ and R_q_ increased more than five times compared to the pure thermoplastic starch film (Table 1). The complex structure of TPS/PH film observed in 3D topographic AFM images can be associated with the highly branched structure of the polysaccharide extract from psyllium husk seeds [1]. According to the one-way ANOVA test, the observed differences in roughness parameters were statistically significant (at the level of significance of α = 0.05). The results of imaging with AFM indicate the usefulness of this method for imaging the impact of additives on the important parameters of the surface of TPS [42].

### 3.2. Surface Characterization

#### 3.2.1. Fourier Transform Infrared (FTIR) Spectroscopy

**Figure 4:**
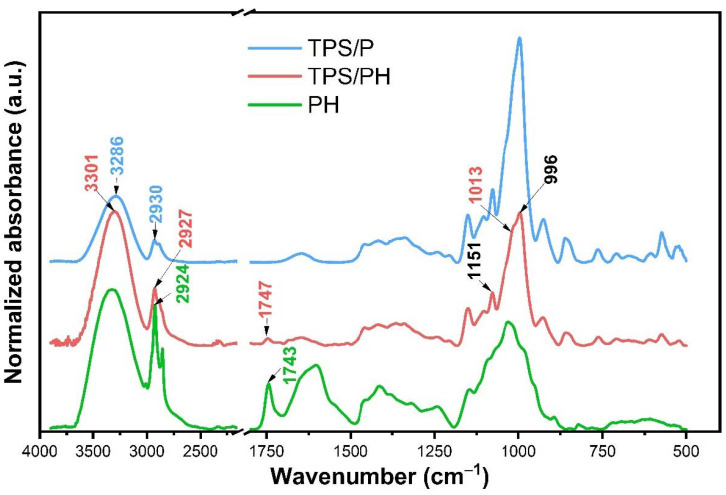
Average FTIR spectra of psyllium husk (PH), native TPS/P, and chemically modified starch composite TPS/PH films. The band positions in black indicate the same values for TPS/P and TPS/PH samples.

The changes in the structure of the films resulting from the interactions between native starch film and the psyllium husk component were determined using FTIR spectroscopy. The FTIR spectra of psyllium husk (PH), native (TPS/P), and modified starch composite films (TPS/PH) are shown in Figure 4. The following regions were distinguished for the functional groups in the tested films: (i) stretching vibrations of the O–H groups, derived from free, intra-, and inter-molecular hydrogen bonds in the native starch structure (3800–3000 cm^−1^), (ii) vibrations extending the C–H groups derived from CH_2_ (2800–3000 cm^−1^), (iii) the δOH bending vibrations at 1646 cm^−1^ associated with the water absorbed in the amorphous part of the starch, (iv) the C–O and C–O–C asymmetric stretching vibrations (1300–1100 cm^−1^) present in natural polysaccharides, and (v) stretching vibrations in the area of 930–730 cm^−1^, characteristic of polysaccharide ring vibrations, attributed to the skeletal vibrations of the pyranose ring in the glucose unit [43]. The intense bands at 1151 and 996 cm^−1^ are attributed to the polysaccharide (1–4)-glycosidic linkage and their corresponding monosaccharides, respectively [44]. A shoulder centered at 1013 cm^−1^ is associated with the C–O stretching vibrations in an anhydroglucose ring.

The broad band at about 3300 cm^−1^ attributed to the –OH vibration is slightly shifted towards higher wave numbers in the case of modified films, which suggests interactions via intermolecular hydrogen bonds between starch-PH and absorbed water molecules in the samples. These findings are in accordance with the previous report obtained for chia-mucilage composite films with starch nanocrystals [45]. In this study [45], the incorporation of starch into the matrix of mucilage film resulted in an increase in the intensity of the OH band and its broadening due to the formation of new intermolecular hydrogen bonds. Psyllium husk consists of soluble (primarily arabinoxylans, AX) and insoluble polysaccharides with plenty of OH groups, which could result in interactions via H-bonds, and which causes the enhanced water-binding capacity of the modified film. The increased intensity of this band could confirm the hydrophilic nature of the TPS/PH film surface discussed in Section 3.2.2. OH groups derived from starch are responsible for forming new functional groups with hydrogen bonds in the PH structure; therefore, the OH-related absorption region becomes more intense and broader [25].

The appearance of an additional band at 1747 cm^−1^ in the spectrum of TPS/PH film likely arises from the C=O group of different sugars (mono- and di-saccharides) that could be related to the soluble fiber from PH (Figure 4, green line). A 4 cm^−1^ shift in the 1743 cm^−1^ band present on the FTIR spectrum of PH to 1747 cm^−1^ in the TPS/PH spectrum may indicate mixing of film components. With the addition of PH, the band intensities at wavenumbers 1200 and 900 cm^−1^ (the C–O and C–O–C vibrations) decreased, which could be related to the changes occurring during the interactions between PH and starch polysaccharides through H-bonding. When the –OH group vibrates more efficiently and/or forms hydrogen bonds, it is more susceptible to present a broader and less intense band [45]. Consequently, PH caused the strengthening of intermolecular hydrogen bonds in the modified films. Krystyjan et al. examined similar starch-based films with psyllium mucilage, but they found no pronounced changes in the FTIR spectra, while there was a marked improvement in the film’s mechanical properties [46]. On the other hand, starch is a heterogeneous material that contains both linear and branched microstructures. Linear chains of amylose exhibit a high tendency to interact through hydrogen bonds [6,47,48]. Previous studies have shown that the film-forming properties of starch depend on its amylose content, and stiff films are formed by linking linear chains by hydrogen bonding [49,50]. FTIR analysis revealed additional intermolecular hydrogen bonding between polysaccharides in starch and PH. Chemically, psyllium husk is a complex polysaccharide predominantly composed of arabinoxylans, which are a highly branched heteroxylan containing arabinose and xylose. In recent years, arabinoxylans, a kind of non-starch polysaccharides, have gained notoriety for their high gel-forming property [50]. Therefore, when the number of hydrogen bonds increases, the mechanical properties are also likely to increase, which was confirmed by the higher film tensile strength discussed in Section 3.4.

#### 3.2.2. Contact Angle Measurements

**Figure 5:**
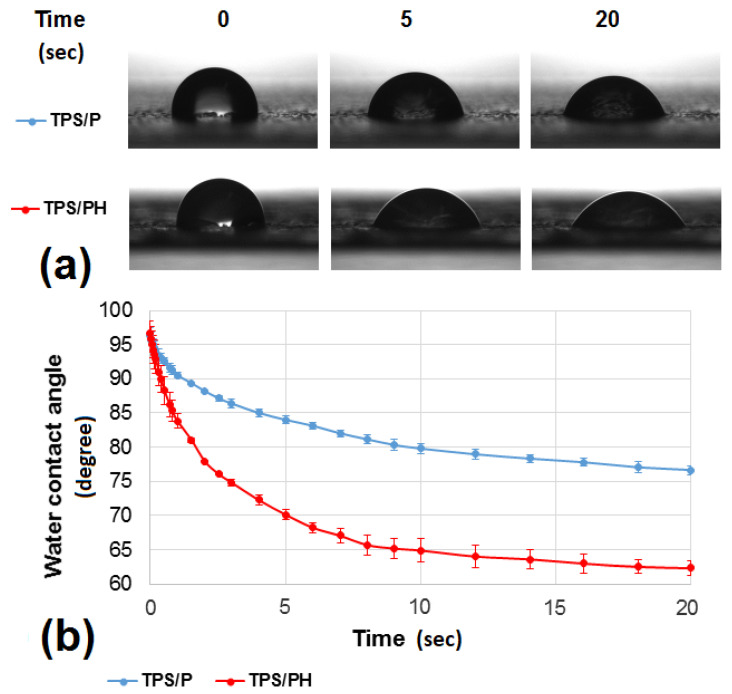
Shape (**a**) and behavior (**b**) of water drops on pure thermoplastic starch film (TPS/P) and thermoplastic starch film with psyllium husk addition (TPS/PH) as a function of time (0–20 s).

The initial value of water contact angles (WCA) measured upon water drop contact with the surface of starch films reached approximately 96°. Simultaneously, a decrease in WCA values was observed over time (Figure 5). Higher change dynamics were observed within the first 5 s, in which the WCA value was reduced to 84.00° for TPS/P and 70.10° for TPS/PH samples. This corresponded to 12.8% and 27.4% of the initial WCA value. At the same time, a significant increase in the contact surface of the water drop with the substrate and a slight decrease in the volume of the applied drop were observed (Table 2). Drop volume decreased on average by 2.5% and 2.0% for the TPS/P and TPS/PH, respectively. In the time interval from 5 to 20 s, WCA dynamics started to decrease and contact angle changes did not exceed 9% for both tested films. Mean values of the drop contact area with the substrate increased from 3.58 to 3.97 mm^2^ and from 4.24 to 4.86 mm^2^ for TPS/P and TPS/PH, respectively. In the case of drop volume, the statistical significance of differences between TPS/P and TPS/PH groups were not found. For the drop contact area at the initial contact angle (t = 0 s), the difference between mean values of tested groups did not differ statistically (*p* = 0.2). However, for times of 5 and 20 s, statistical significance of differences between TPS/P and TPS/PH samples was observed. Obtained *p*-values amounted to 0.0002 and 0.00005, respectively.

It is well-known that the water contact angle will decrease with the increasing surface hydrophilicity, thus being a primary indicator for hydrophobic characterization of polymer materials [51]. The contact angle of biopolymers ranges from 0° (complete spreading of the liquid onto the solid surface) to 180° (an unrealistic limit of absolutely no wetting) [52]. A biopolymer can be considered hydrophilic when its contact angle is lower than 90°, and hydrophobic when the contact angle is higher than 90° [51,53].

Initial contact angle values are primarily dependent on the chemical nature of the surface, and high initial WCA values suggest a slightly hydrophobic nature of the films. According to Białopiotrowicz [54], during the film-forming process, the most hydrophobic and branched parts of the amylopectin chain are directed outwards in the form of a gel film, which determines the initial wettability of the material. This explains the similar initial contact angle values obtained for both kinds of starch films. Nevertheless, as a result of steric interactions, there may still be polar domains formed by functional groups between carbon chains [54]. In the case of pure starch films (TPS/P), polar groups are mainly derived from glucose, and in the case of psyllium husk films (TPS/PH), also from mono- and di-saccharides rich in hydroxyl groups. It can result in greater wetting dynamics, which was observed for the starch film with the addition of psyllium husk, and it was also confirmed in [55]. The author notes that water contact angles of the films decreased, as a function of time, due to the reorientation of polar groups on the film surface.

Moreover, in the case of absorbing substrates, including those based on starch, complete stability between the drop applied and the test material is generally not achieved [56]. As some authors point out [57,58,59], this is the result of partial absorption of the liquid into the substrate, which reduces the contact angle. Typically, liquid absorption increases and the contact angle decreases with increasing porosity [60,61], pore size [62], and material permeability [59]. Various arrangements and shapes of the pores may also be a factor influencing the penetration of droplets into the material [57]. In the case of the analyzed films, the change in the volume of the applied drop over time was small and comparable for both materials. This means that the physical inhomogeneity of the surface of starch films did not have a crucial influence on the observed dynamics of WCA changes.

The hydrophilic nature of biodegradable films with the addition of psyllium husk was also demonstrated by Ahmadi et al. [19]. However, the results of research on psyllium husk-based films (made without thermoplastic starch), with a different content of glycerol, showed much lower water contact angle values. On the other hand, Zhang et al. [27] investigated the effect of psyllium husk gum on the properties of whey protein composites and observed much higher contact angles (about 98–128°).

#### 3.2.3. Water Sorption Isotherms

**Figure 6:**
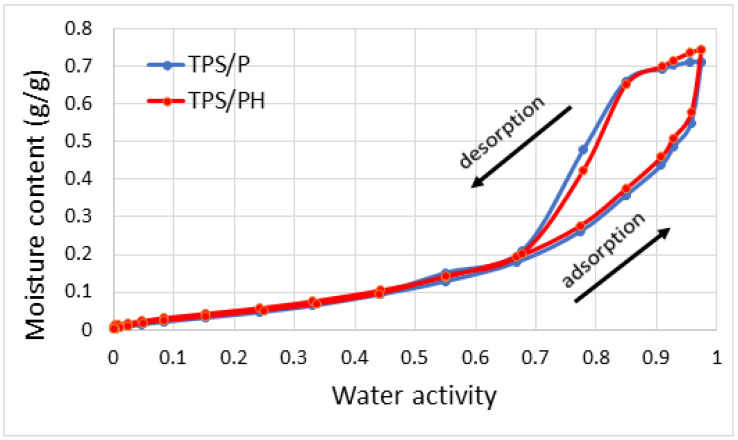
Water sorption/desorption isotherms of the analyzed thermoplastic starch films.

Figure 6 presents water vapor sorption isotherms of TPS/P and TPS/PH films. The S-shape of the isotherms indicates that they are type II of the Brunauer–Emmett–Teller classification [60]. This curve shape is observed for many carbohydrate polymer-based films [62,63,64]. Sorption isotherms were characterized by the presence of a slight inflection at low water vapor pressures (a_w_ ≈ 0.1), a more or less inclined middle part, a_w_ from ≈0.1 to 0.7, and a rapid increase in the amount of adsorbed water vapor with a_w_ ≈ 0.7. Adsorption and desorption processes were not fully reversible, revealing hysteresis at 0.68 a_w_ values. The observed hysteresis loops belong to the H2 type according to the IUPAC classification, without a clear saturation effect at high a_w_ values [65]. The presence of hysteresis was explained by Caurie [66], who stated that the desorption isotherm occurs at higher energy levels compared to the adsorption isotherm and that the energy difference may result from changes in the adsorbent structure and the formation of pores, microcracks, and fissures. Hysteresis may also reflect conformational rearrangements, which alter the accessibility of energetically favorable polar sites and influence sorption processes and water movement.

Quantitative differences in water vapor adsorption were expressed by changes in the specific surface area (SSA) and average adsorption energy (E_av_). A higher mean value of SSA (212.00 ± 0.94 m^2^/g) was obtained for TPS/PH. This suggests that the TPS/PH surface consists of more reactive sites for water vapor molecules compared to TPS/P. These sites can be hydroxyl, phenol, or carboxyl groups. In the case of TPS/P film, SSA equaled 205.12 ± 2.67 m^2^/g. The observed differences between the variants were statistically significant at the level of significance of α= 0.05. A higher mean value of adsorption energy (E_av_) was obtained for THPS/PH (1.84 ± 0.02 ΔE/RT). This may be attributed to the formation of strong hydrogen bonds with polar functional groups, whose presence was proven by FTIR measurements. In the case of TPS/P, the adsorption energy was 1.36 ± 0.01 ΔE/RT. The performed statistical analysis showed that the observed differences were significant (*p* < 0.05).

The adsorption branch of the isotherms in the region of capillary condensation was also used for fractal dimension analysis. For flat surfaces, the fractal dimension (D) takes the value of 2 and tends to the value of 3 with increasing surface roughness [67]. In our work, D equaled 2.16 and 2.21 for TPS/P and TPS/PH samples, respectively. The obtained results showed that starch films modified by psyllium husk were characterized by higher surface heterogeneity, which was also proven by AFM measurements.

### 3.3. Barrier Properties

#### 3.3.1. Water Vapor Permeability (WVP)

Water is responsible for texture changes in food that affect food quality and stability. Therefore, food packaging is designed to block moisture transfer between food and the environment, hence requiring lower water vapor permeability of packaging films for practical use [15].

The TPS/PH films exhibited a significant increase in WVP compared to TPS/P films (Table 3). However, this result was in contrast to the conclusions drawn by Sukhija et al. [25], who showed a tendency to reduce the WVP value of starch-based films by incorporation of psyllium husks to the film-forming solution. Likewise, a decrease in the WVP value was reported in the case of films made with the addition of psyllium husk flour in comparison to those made with psyllium husk seeds [1].

The authors of the abovementioned works emphasized the significant role of plasticizers in increasing the WVP of psyllium husk films. It should also be noted that the different observations can be caused by the diversities in chemical composition in film-forming solution or cracks on their structures which have a large impact on the WVP of the films [19]. However, factors such as film thickness or type of extraction method, as well as the reactions between polymer functional groups, also affect the WVP value [27]. Moreover, a reduction of the WVP value of films containing psyllium husk gum (PHG—a polysaccharide fraction extracted from psyllium seeds) was shown, which was also supported by studies with similar results obtained by other authors [68], who explained that the low WVP value is due to the branched and compact structure of psyllium hydrocolloid that prevents the penetration and transition of water molecules. As a result of research in [68], a lower WVP coefficient was obtained for films made of thermoplastic starch (3% *w*/*v*), WVP = 2.18 × 10^−10^, compared to WVP = 1.45 × 10^−10^ for films made of 100% of psyllium husk gum. The reason for that may be the higher thickness of the TPS films (equal to 0.13 mm) compared to 0.08 mm for films made of psyllium husk gum. On the other hand, the psyllium husk gum films showed a much lower tensile strength than films made of pure thermoplastic starch, and in [1], the reduction of WVP goes along with better mechanical properties of the film made only with psyllium husk flour.

#### 3.3.2. UV-Vis Barrier Properties (Opacity and Light Transmittance)

The film-forming components affected the thickness of the films and their optical properties [29]. It is likely that due to the retraction of the starch gel during drying of clear films, there was an increase in amylopectin content at the expense of amylose. Amylopectin promotes the formation of less heterogeneous and denser films and thus resulted in the creation of thinner films [69].

**Figure 7:**
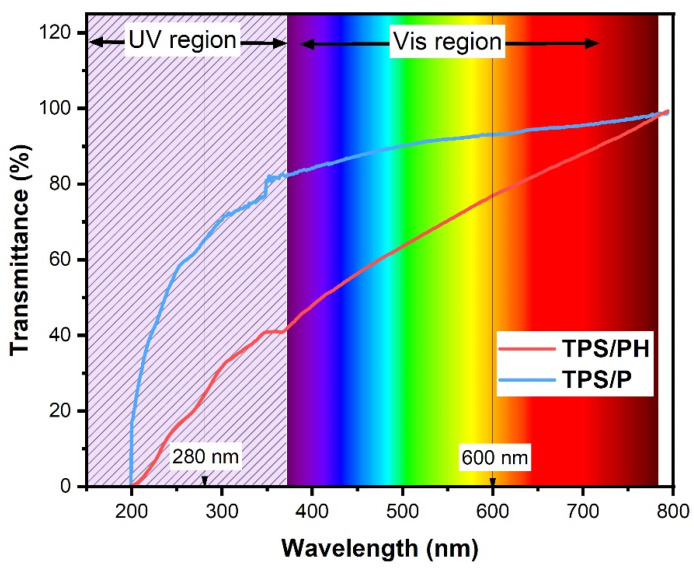
The UV-Vis wavelength transmission curves of TPS/P (blue line) and TPS/PH films (red line). The background shows the colors of light in the UV-visible spectrum.

The UV-Vis transmittance patterns recorded for TPS/P and TPS/PH film samples are presented in Figure 7, and the values of transmittance and opacity at 280 and 600 nm are collected in Table 3. Adding PH led to a significant decrease in the transmittance of UV (T_r280_) and visible light (Tr_600_), while opacity was increased [70]. The opacity of composite film at 600 nm increased with the addition of PH and was 43% higher than in the pure native starch film. Previous studies reported on the increasing opacity of biocomposites with increasing fiber content. The TPS/PH film sample exhibited low light transmission in UV light, especially at 280 nm. Films with PH show lower percent transmittance compared with the non-modified film, suggesting that TPS/PH films could have good barrier properties to UV light, a potent lipid-oxidizing agent in food systems. 

### 3.4. Tensile Properties

#### 3.4.1. Tensile Tests Analysis

**Figure 8:**
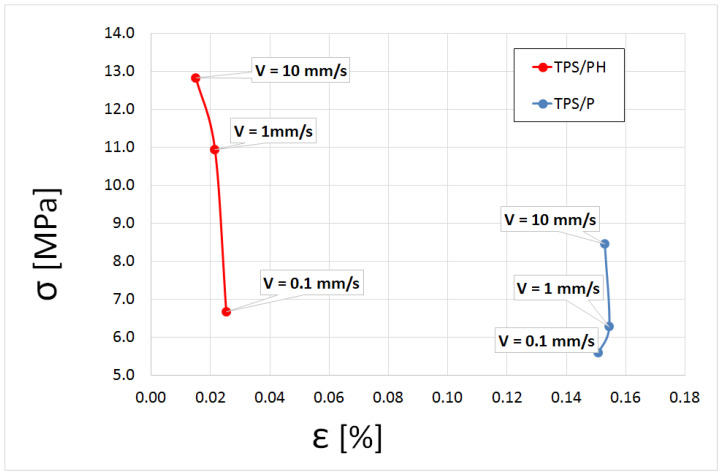
The relationship between failure stress, σ (MPa), and failure strain, ε (%), at different deformation velocities.

According to [71], tensile strength and elongation are two very important properties in packaging materials because they reflect the durability of films and their ability to enhance the mechanical integrity of food. Figure 8 shows the relationship between failure stress and failure strain at different deformation velocities (V = 0.1, V = 1, V = 10 mm/s). For the TPS/P film, as the deformation velocity increases, the failure point moves towards a higher stress, with strain values changing inconsiderably (statistically significant differences between the mean values of failure strain for the tested deformation velocities were not found). The addition of psyllium husk (TPS/PH films) resulted in an increase of the failure stress with a simultaneous decrease in the failure strain at the same deformation velocities (Figure 8). The mean values of failure strain for the tested deformation velocities were 86% lower for the psyllium husk-containing film than for the pure film, in contrast to the failure stress where the addition of PH increased by an average of 48% compared to the pure film in the range of the tested deformation velocities. The very small change of the failure strain in the tested range of deformation velocities may indicate that the damage of the film was caused by exceeding a specified strain value. This may prove the validity of the maximum strain criterion for the thermoplastic biodegradable film for deformation velocities in the range of 0.1–10 mm/s. Ahmadi et al. [19], who carried out tests on films made on the basis of psyllium husk (without TPS) with a similar glycerol content, at V = 50 mm/min, obtained a tensile strength of about 10 MPa and an elongation of 30%. In the same study, a decrease in tensile strength was observed with the higher content of this plasticizer. Glycerol, a small-molecular-sized plasticizer, easily diffuses into starch polymer chains to disrupt their hydrogen bond interaction [72]. It can be suspected that the higher tensile strength of the TPS film with the addition of psyllium husk may be related to the presence of more hydrogen bonds, which was confirmed by FTIR spectroscopy results, but this is also in agreement with other studies [24,73]. Chinma et al. [73] found that the higher tensile strength of composite film may be anticipated as a result of the addition of psyllium husk, which might cause the formation of new bonds between starch and protein matrixes due to its hydrocolloid nature. In our study, such interactions had a minor role due to the very low PH content (1%) in the films, and, presumably, the H-bond formation between the components and various amounts of water content affected the mechanical properties of TPS/PH films.

#### 3.4.2. Fracture Surface Analyses (SEM)

**Figure 9:**
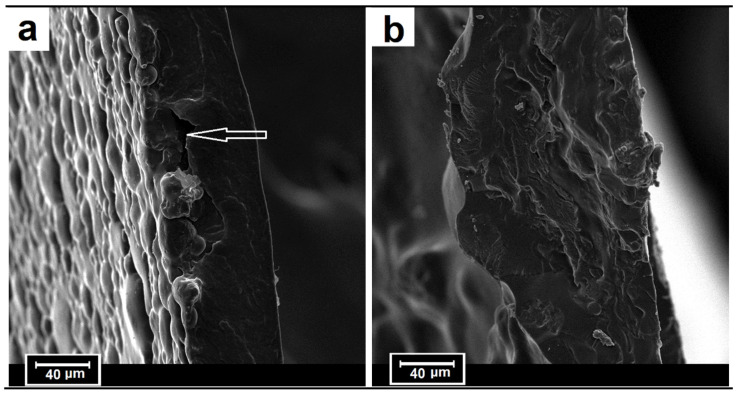
SEM observations of the fractures: (**a**) TPS/P and (**b**) TPS/PH.

Observations of fracture morphology showed no differences between the samples after tensile tests at different speeds. Representative fracture structures of TPS/P and TPS/PH are shown in Figure 9. TPS/P fractures had a smooth surface (Figure 9a). Some discontinuities in the internal structure of the TPS/P film (shown by the white arrow) are present, which may be the reason for the lower tensile strength. TPS/PH samples have a dense morphology of the fractures with no visible cavities, but with a visible rough surface with brittle fracture characteristics (Figure 9b). Additionally, the appearance of microcracks was noted in the fractures of the TPS/P film under the surface of the bumps caused by the starch granules. Together with the microcracks on the surface (Figure 2b), they indicate the brittle adhesion structure of the gelatin phase around starch granules [37].

## 4. Conclusions

In the present study, thermoplastic starch/psyllium husk composite films were manufactured by incorporating 1% of psyllium husk into a starch matrix using the casting method. The effect of PH addition on the TPS films was investigated by an examination of the microstructure, barrier properties, and mechanical resistance of the modified films. The surface parameters morphology and wettability were analyzed by atomic force microscopy (AFM) and contact angle measurements. The barrier properties were studied by determining water vapor permeability. Micro-tensile tests analyzed the mechanical properties under various static loading conditions. The visible effect of PH addition was manifested in a more uniform structure of TPS/PH films with fewer “round granules” and without microcracks. The presence of additional hydrogen bonds in the modified films improved their tensile strength (for deformation velocities in the range of 0.1–10 mm/s). This implies that psyllium husk promotes starch gelatinization and causes a decrease in the failure strain by 86% and an increase in the failure stress by 48% compared to pure films. A greater number and availability of hydroxyl groups derived from mono- and di-saccharides increased the wetting dynamics: (i) a faster decrease in the WCA value from 27.4% compared to 12.8% in pure TPS films within the first 5 s was observed, and (ii) the VWP value increased by 35% for the films with the PH addition. The modified films displayed the opacity at 600 nm, 43% higher than in the pure starch film, and lower transmittance, suggesting effectively improving barrier properties to UV light, a potent lipid-oxidizing agent in food systems.

Moreover, Fourier transform infrared spectroscopy results confirmed the incorporation of PH into TPS films by forming hydrogen bonds between polysaccharides arising from starch and PH. The psyllium husk can provide an appropriate matrix for manufacturing the films blended with other polymers applied in the food packaging and coatings industry. Only the problem of excessive water absorption of the modified films may require improvement in future studies. The addition of a different amount of psyllium husk is planned, and the ground form of psyllium husk, the so-called psyllium flour, should improve the film’s structure and reduce water penetration. Modifying the amount of plasticizer (glycerol) and using additional ingredients limiting water absorption (e.g., beeswax, nano-silica) should bring beneficial results.

## Figures and Tables

**Table 1:** Roughness parameters of thermoplastic starch films.

Sample	Thickness (mm)	R_a_ (nm)	R_q_ (nm)	R_p_ (nm)	R_v_ (nm)
TPS/P	0.09 ± 0.01	22.15 ± 4.40 ^a^	28.23 ± 5.90 ^a^	82.91 ± 32.42 ^a^	60.23 ± 15.54 ^a^
TPS/PH	0.16 ± 0.02	119.99 ± 30.48 ^b^	145.09 ± 34.23 ^b^	291.32 ± 92.33 ^b^	271.65 ± 86.25 ^b^

R_a_—Arithmetical mean deviation of the roughness profile ± SD, R_q_—root-mean-square deviation of the roughness profile ± SD, R_p_—maximum peak height of the roughness profile ± SD, R_v_—maximum valley depth of the roughness profile ± SD. Different letters indicate significant differences between the values at the level of significance, α = 0.05, one-way ANOVA, Tukey’s HSD test. Values are mean ± SD.

**Table 2:** Drop volume and surface of contact of the drop with TPS/P and TPS/PH films as a function of time (0–20 s). Values are mean ± SD.

	TPS/P			TPS/PH		
Time (s):	0	5	20	0	5	20
Drop volume (mm^3^)	1.99 (±0.06)	1.94 (±0.08)	1.90 (±0.10)	1.97 (±0.06)	1.93 (±0.07)	1.90 (±0.08)
Contact surface (mm^2^)	2.75 (±0.07)	3.58 (±0.27)	3.97 (±0.27)	2.87 (±0.19)	4.24 (±0.20)	4.86 (±0.25)

**Table 3:** Water vapor permeability (WVP) (g m^−1^ s^−1^ Pa^−1^), light transmittance (%), and transparency value of the prepared films.

Sample	WVP (g m^−1^ s^−1^ Pa^−1^)	T_r280_ (%)	T_r600_ (%)	Opacity (AU/mm)
TPS/P	3.38 × 10^−10^ ± 8.57 × 10^−12 a^	69.39 ± 2.74 ^a^	94.62 ± 0.98 ^a^	0.28 ± 0.05 ^a^
TPS/PH	4.55 × 10^−10^ ± 1.74 × 10^−12 b^	23.87 ± 1.59 ^b^	78.97 ± 1.29 ^b^	0.65 ± 0.04 ^b^

T_r280_, light transmittance at 280 nm; T_r600_, light transmittance at 600 nm. Data are shown as mean ± standard deviation. Means in a column with the same superscripts are not significantly different (*p* < 0.05).

## Data Availability

Data is contained within the article.

## Abbreviations

**Nomenclature and Greek Symbols**	
E_av_	average adsorption energy (∆E/RT)
∆E	difference between energy of adsorption (E_a_) and condensation (E_c_) of water vapor
R	universal gas constant (J·mol^−1^·K^−1^)
T	temperature (K)
D	surface fractal dimension
A	effective area of films (m^2^)
∆m	the weight change of the permeation vessel (g)
t	time (s)
O_600_	transparency (AU/mm)
T_600_	transmittance at 600 nm (%)
F_max_	maximum force (N)
R_a_	arithmetical mean deviation of the roughness profile (nm)
R_q_	root-mean-square deviation of the roughness profile (nm)
R_p_	maximum peak height of the roughness profile (nm)
R_v_	maximum valley depth of the roughness profile (nm)
σ_f_	failure stress (MPa)
ε_f_	failure strain (%)
V	deformation velocities (mm/s)
δOH	OH bending vibrations
H-bonds	hydrogen bonding
**Abbreviations**	
TPS/P	pure thermoplastic starch film
TPS/PH	thermoplastic starch film + psyllium husk
PH	psyllium husk
UV	ultraviolet
WVP	water vapor permeability (g·m^−1^ s^−1^ Pa^−1^)
RH	relative humidity (%)
WCA	water contact angle (deg)
SSA	specific surface area (m^2^/g)
WVTR	water vapor transmission (g/s)
AX	arabinoxylans

## References

[1] Tóth A., Halász K. (2019). Characterization of edible biocomposite films directly prepared from psyllium seed husk and husk flour. Food Packag. Shelf Life.

[2] EU Directive 2019/904 of the European Parliament and of the Council of 5 June 2019 on the Reduction of the Impact of Certain Plastic Products on the Environment (Text with EEA Relevance) PE/11/2019/REV/. http://data.europa.eu/eli/dir/2019/904/oj.

[3] Single-Use Plastics: New EU Rules to Reduce Marine Litter. https://ec.europa.eu/commission/presscorner/detail/en/MEMO_18_3909.

[4] García-Guzmán L., Cabrera-Barjas G., Soria-Hernández C.G., Castaño J., Guadarrama-Lezama A.Y., Llamazares S.R. (2022). Progress in Starch-Based Materials for Food Packaging Applications. Polysaccharides.

[5] Temesgen S., Rennert M., Tesfaye T., Nase M. (2021). Review on Spinning of Biopolymer Fibers from Starch. Polymers.

[6] Jiang T., Duan Q., Zhu J., Liu H., Yu L. (2019). Starch-based biodegradable materials: Challenges and opportunities. Adv. Ind. Eng. Polym. Res..

[7] Dang K.M., Yoksan R. (2021). Thermoplastic starch blown films with improved mechanical and barrier properties. Int. J. Biol. Macromol..

[8] Avérous L., Boquillon N. (2004). Biocomposites based on plasticized starch: Thermal and mechanical behaviors. Carbohydr. Polym..

[9] Tosif M., Najda A., Bains A., Zawiślak G., Maj G., Chawla P. (2021). Starch–Mucilage Composite Films: An Inclusive on Physicochemical and Biological Perspective. Polymers.

[10] Ren J., Dang K.M., Pollet E., Avérous L. (2018). Preparation and Characterization of Thermoplastic Potato Starch/Halloysite Nano-Biocomposites: Effect of Plasticizer Nature and Nanoclay Content. Polymers.

[11] Girijappa Y.G.T., Rangappa S.M., Parameswaranpillai J., Siengchin S. (2019). Natural Fibers as Sustainable and Renewable Resource for Development of Eco-Friendly Composites: A Comprehensive Review. Front. Mater..

[12] Yang J., Tang K., Qin G., Chen Y., Peng L., Wan X., Xiao H., Xia Q. (2017). Hydrogen bonding energy determined by molecular dynamics simulation and correlation to properties of thermoplastic starch films. Carbohydr. Polym..

[13] Basiak E., Lenart A., Debeaufort F. (2018). How Glycerol and Water Contents Affect the Structural and Functional Properties of Starch-Based Edible Films. Polymers.

[14] Gołacki K., Stropek Z., Kołodziej P., Gładyszewska B., Zaremba M., Rejak A. (2014). Effect of additives on strength characteristics of a biodegradable starch film. Przem. Chem..

[15] Khan B., Niazi M.B.K., Samin G., Jahan Z. (2016). Thermoplastic Starch: A Possible Biodegradable Food Packaging Material-A Review. J. Food Process Eng..

[16] Stropek Z., Gołacki K., Kołodziej P., Gładyszewska B., Samociuk W., Rejak A. (2014). Effect of polyvinyl alcohol and keratin on stress relaxation course in thermoplastic starch. Przem. Chem..

[17] Krystyjan M., Khachatryan G., Khachatryan K., Konieczna-Molenda A., Grzesiakowska A., Kuchta-Gładysz M., Kawecka A., Grzebieniarz W., Nowak N. (2022). The Functional and Application Possibilities of Starch/Chitosan Polymer Composites Modified by Graphene Oxide. Int. J. Mol. Sci..

[18] Kwaśniewska A., Chocyk D., Gładyszewski G., Borc J., Świetlicki M., Gładyszewska B. (2020). The Influence of Kaolin Clay on the Mechanical Properties and Structure of Thermoplastic Starch Films. Polymers.

[19] Ahmadi R., Kalbasi-Ashtari A., Oromiehie A., Yarmand M.-S., Jahandideh F. (2012). Development and characterization of a novel biodegradable edible film obtained from psyllium seed (*Plantago ovata* Forsk). J. Food Eng..

[20] Patil B.S., Mastiholimath V.S., Kulkarni A.R. (2011). Development and evaluation of psyllium seed husk polysaccharide based wound dressing films. Orient. Pharm. Exp. Med..

[21] Hussain M.A., Muhammad G., Jantan I., Bukhari S.N.A. (2015). Psyllium Arabinoxylan: A Versatile Biomaterial for Potential Medicinal and Pharmaceutical Applications. Polym. Rev..

[22] Debeaufort F., Martin-Polo M., Voilley A. (1993). Polarity Homogeneity and Structure Affect Water Vapor Permeability of Model Edible Films. J. Food Sci..

[23] Fischer M.H., Yu N., Gray G.R., Ralph J., Anderson L., Marlett J.A. (2004). The gel-forming polysaccharide of psyllium husk (*Plantago ovata* Forsk). Carbohydr. Res..

[24] Sukhija S., Singh S., Riar C.S. (2016). Analyzing the effect of whey protein concentrate and psyllium husk on various characteristics of biodegradable film from lotus (*Nelumbo nucifera*) rhizome starch. Food Hydrocoll..

[25] Sukhija S., Singh S., Riar C.S. (2018). Physical, Mechanical, Morphological, and Barrier Properties of Elephant Foot Yam Starch, Whey Protein Concentrate and psyllium Husk Based Composite Biodegradable Films. Polym. Compos..

[26] Sukhija S., Singh S., Riar C.S. (2019). Development and characterization of biodegradable films from whey protein concentrate, psyllium husk and oxidized, crosslinked, dual-modified lotus rhizome starch composite. J. Sci. Food Agric..

[27] Zhang X., Zhao Y., Li Y., Zhu L., Fang Z., Shi Q. (2020). Physicochemical, mechanical and structural properties of composite edible films based on whey protein isolate/psyllium seed gum. Int. J. Biol. Macromol..

[28] Karel M., Karel M., Fennema O.R., Lund D.B. (1975). Water activity and food preservation. Physical Principles of Food Preservation—Principles of Food Science—Part 2.

[29] Basiak E., Lenart A., Debeaufort F. (2017). Effect of starch type on the physico-chemical properties of edible films. Int. J. Biol. Macromol..

[30] Brunauer S., Emmett P.H., Teller E. (1938). Adsorption of Gases in Multimolecular Layers. J. Am. Chem. Soc..

[31] Jozefaciuk G., Lukowska M., Szerement J. (2013). Determination of Energetic and Geometric Properties of Plant Roots Specific Surface from Adsorption/Desorption Ishoterm. Am. J. Plant Sci..

[32] Sokołowska Z., Hajnos M., Borówko M., Sokołowski S. (1999). Adsorption of Nitrogen on Thermally Treated Peat Soils: The Role of Energetic and Geometric Heterogeneity. J. Colloid Interface Sci..

[33] Souza A., Benze R., Ferrão E., Ditchfield C., Coelho A., Tadini C. (2011). Cassava starch biodegradable films: Influence of glycerol and clay nanoparticles content on tensile and barrier properties and glass transition temperature. LWT.

[34] ASTM (2016). Standard test methods for water vapor transmission of materials. Standards designations: E96/E96M-16. Annual Book of ASTM Standards.

[35] Han J.H., Floros J.D. (1997). Casting Antimicrobial Packaging Films and Measuring Their Physical Properties and Antimicrobial Activity. J. Plast. Film Sheeting.

[36] Cheng Y., Sun C., Zhai X., Zhang R., Zhang S., Sun C., Wang W., Hou H. (2021). Effect of lipids with different physical state on the physicochemical properties of starch/gelatin edible films prepared by extrusion blowing. Int. J. Biol. Macromol..

[37] Cheng Y., Gao S., Wang W., Hou H., Lim L.-T. (2021). Low temperature extrusion blown ε-polylysine hydrochloride-loaded starch/gelatin edible antimicrobial films. Carbohydr. Polym..

[38] Huntrakul K., Yoksan R., Sane A., Harnkarnsujarit N. (2020). Effects of pea protein on properties of cassava starch edible films produced by blown-film extrusion for oil packaging. Food Packag. Shelf Life.

[39] Wang W., Zhang H., Jia R., Dai Y., Dong H., Hou H., Guo Q. (2018). High performance extrusion blown starch/polyvinyl alcohol/clay nanocomposite films. Food Hydrocoll..

[40] Acosta S., Jiménez A., Cháfer M., González-Martínez C., Chiralt A. (2015). Physical properties and stability of starch-gelatin based films as affected by the addition of esters of fatty acids. Food Hydrocoll..

[41] Gutiérrez T.J., Álvarez V. (2018). Bionanocomposite films developed from corn starch and natural and modified nano-clays with or without added blueberry extract. Food Hydrocoll..

[42] Kwaśniewska A., Świetlicki M., Prószyński A., Gładyszewski G. (2021). The Quantitative Nanomechanical Mapping of Starch/Kaolin Film Surfaces by Peak Force AFM. Polymers.

[43] Warren F.J., Gidley M.J., Flanagan B.M. (2016). Infrared spectroscopy as a tool to characterise starch ordered structure—A joint FTIR-ATR, NMR, XRD and DSC study. Carbohydr. Polym..

[44] Nikonenko N.A., Buslov D.K., Sushko N.I., Zhbankov R.G. (2000). Investigation of stretching vibrations of glycosidic linkages in disaccharides and polysaccharides with use of IR spectra deconvolution. Biopolymers.

[45] Mujtaba M., Koç B., Salaberria A.M., Ilk S., Duman D.C., Akyüz L., Cakmak Y.S., Kaya M., Khawar K.M., Labidi J. (2019). Production of novel chia-mucilage nanocomposite films with starch nanocrystals; An inclusive biological and physicochemical perspective. Int. J. Biol. Macromol..

[46] Krystyjan M., Khachatryan G., Ciesielski W., Buksa K., Sikora M. (2017). Preparation and characteristics of mechanical and functional properties of starch/*Plantago psyllium* seeds mucilage films. Starch.

[47] Jenkins P.J., Donald A.M. (1995). The influence of amylose on starch granule structure. Int. J. Biol. Macromol..

[48] Lourdin D., Della Valle G., Colonna P. (1995). Influence of amylose content on starch films and foams. Carbohydr. Polym..

[49] Tarique J., Sapuan S.M., Khalina A. (2021). Effect of glycerol plasticizer loading on the physical, mechanical, thermal, and barrier properties of arrowroot (*Maranta arundinacea*) starch biopolymers. Sci. Rep..

[50] Singh A., Benjakul S., Prodpran T., Nuthong P. (2021). Effect of Psyllium (*Plantago ovata* Forks) Husk on Characteristics, Rheological and Textural Properties of Threadfin Bream Surimi Gel. Foods.

[51] Li X., Yan J., Yu T., Zhang B. (2022). Versatile nonfluorinated superhydrophobic coating with self-cleaning, anti-fouling, anti-corrosion and mechanical stability. Colloids Surf. A Physicochem. Eng. Asp..

[52] Li X.W., Wang H.X., Shi T., Zhang C.W., Jiang X.N., Zhou X.G., Li C. (2022). Efficient preparation and anticorrosion mechanism of superhydrophobic 7075 aviation aluminum alloy. Rare Met. Mater. Eng..

[53] Jouki M., Khazaei N., Ghasemlou M., HadiNezhad M. (2013). Effect of glycerol concentration on edible film production from cress seed carbohydrate gum. Carbohydr. Polym..

[54] Białopiotrowicz T. (2003). Wettability of starch gel films. Food Hydrocoll..

[55] Wiącek A.E. (2015). Effect of surface modification on starch biopolymer wettability. Food Hydrocoll..

[56] Krainer S., Hirn U. (2021). Contact angle measurement on porous substrates: Effect of liquid absorption and drop size. Colloids Surfaces A Physicochem. Eng. Asp..

[57] Gambaryan-Roisman T. (2014). Liquids on porous layers: Wetting, imbibition and transport processes. Curr. Opin. Colloid Interface Sci..

[58] Espín L., Kumar S. (2015). Droplet spreading and absorption on rough, permeable substrates. J. Fluid Mech..

[59] Susana L., Campaci F., Santomaso A.C. (2012). Wettability of mineral and metallic powders: Applicability and limitations of sessile drop method and Washburn’s technique. Powder Technol..

[60] André V., Zosel A.D. (1994). Dynamic wetting on porous and non porous substrates. Influence of surface tension, viscosity and porosity. Ber. Bunsenges. Phys. Chem..

[61] Letellier P., Mayaffre A., Turmine M. (2007). Drop size effect on contact angle explained by nonextensive thermodynamics. Young’s equation revisited. J. Colloid Interface Sci..

[62] Pan Y., Huang Z., Li T., Xu X., Chen X., Guo X. (2021). Pore structure characteristics and evaluation of lacustrine mixed fine-grained sedimentary rocks: A case study of the Lucaogou Formation in the Malang Sag, Santanghu Basin, Western China. J. Pet. Sci. Eng..

[63] Brunauer S., Deming L.S., Deming W.E., Teller E. (1940). On a Theory of the van der Waals Adsorption of Gases. J. Am. Chem. Soc..

[64] Cheviron P., Gouanvé F., Espuche E. (2016). Preparation, characterization and barrier properties of silver/montmorillonite/starch nanocomposite films. J. Membr. Sci..

[65] Ayadi F., Dole P. (2011). Stoichiometric interpretation of thermoplastic starch water sorption and relation to mechanical behavior. Carbohydr. Polym..

[66] Caurie M. (2007). Hysteresis phenomenon in foods. Int. J. Food Sci. Technol..

[67] Liu K., Ostadhassan M., Jang H.W., Zakharova N.V., Shokouhimehr M. (2021). Comparison of fractal dimensions from nitrogen adsorption data in shale via different models. RSC Adv..

[68] Askari F., Sadeghi E., Mohammadi R., Rouhi M., Taghizadeh M., Shirgardoun M.H., Kariminejad M. (2018). The physicochemical and structural properties of psyllium gum/modified starch composite edible film. J. Food Process. Preserv..

[69] Stading M., Rindlav-Westling Å., Gatenholm P. (2001). Humidity-induced structural transitions in amylose and amylopectin films. Carbohydr. Polym..

[70] Zhu Y., Ye T., Jiang S., Lin L., Lu J. (2022). Effects of psyllium husk powder on the gel properties of silver carp (*Hypophthalmichthys molitrix*) surimi. J. Food Process. Preserv..

[71] Pranoto Y., Salokhe V.M., Rakshit S.K. (2005). Physical and antibacte rial properties of alginate-based edible film incorporated with garlic oil. Food Res. Int..

[72] Daudt R., Avena-Bustillos R., Williams T., Wood D., Külkamp-Guerreiro I., Marczak L., McHugh T. (2016). Comparative study on properties of edible films based on pinhão (*Araucaria angustifolia*) starch and flour. Food Hydrocoll..

[73] Chinma C.E., Ariahu C.C., Abu J.O. (2011). Development and characterization of cassava starch and soy protein concentrate based edible films. Int. J. Food Sci. Technol..

